# Human Leukocyte Antigen Determinants in Myocarditis and Dilated Cardiomyopathy

**DOI:** 10.1007/s11897-026-00761-0

**Published:** 2026-04-24

**Authors:** Nihit Goli, Jennifer Wilcox, John Barnard, W. H. Wilson Tang

**Affiliations:** 1 Heart, Blood, and Kidney Research, Cleveland Clinic Research, Cleveland Clinic, Cleveland, OH USA; 2 Department of Quantitative Health Sciences, Cleveland Clinic Research, Cleveland Clinic, Cleveland, OH USA; 3 Cleveland Clinic Lerner College of Medicine of Case Western Reserve University, Cleveland, OH USA; 4 Kaufman Center for Heart Failure Treatment and Recovery, Heart Vascular and Thoracic Institute, Cleveland Clinic, 9500 Euclid Avene, Desk J3-4, Cleveland, OH 44195 USA

**Keywords:** Myocarditis, Dilated cardiomyopathy, Human leukocyte antigen, Autoimmune cardiomyopathy, Viral myocarditis, Immune-mediated heart failure

## Abstract

**Purpose of Review:**

Myocarditis, defined as inflammation of heart muscle, can be triggered by viral infection or have an autoimmune origin. In some cases, it progresses to dilated cardiomyopathy (DCM) and heart failure. The human leukocyte antigen (HLA) system is important in adaptive immunity by influencing antigen presentation and autoimmunity. This review intends to clarify the role of HLA polymorphisms in the progression, resistance, and outcomes of myocarditis and DCM.

**Recent Findings:**

Evidence indicates that HLA is linked to myocarditis and DCM through familial aggregation, myocardial HLA upregulation, circulating cardiac autoantibodies, candidate association studies, genome-wide association signals at chromosome 6p21, and transgenic models demonstrating HLA-restricted disease susceptibility. HLA alleles influence viral clearance, molecular mimicry, autoimmune priming, cytokine signaling, and pathogen-specific autoantibody production. Translation of this evidence into clinical practice has been limited by modest effect sizes, population variability, and the lack of genotype-guided therapies.

**Summary:**

Using HLA as a risk-stratification tool can allow more accurate surveillance and immunomodulatory strategies for myocarditis and DCM. Future research priorities should include multi-ethnic HLA studies, viral epitope mapping, integration of immune profiling, and HLA-stratified clinical trials.

## Introduction

Myocarditis, which is inflammation of the heart muscle, is often triggered by viral infections and autoimmunity [1–6]. An initial immune response against a viral pathogen can shift to attack cardiac self-antigens, turning an acute infection-triggered injury into a chronic autoimmune process [4, 7, 8]. While it can resolve without long-term consequences, in some cases myocarditis progresses to dilated cardiomyopathy (DCM), a chronic disease state [4–7, 9, 10].

The Human Leukocyte Antigen (HLA) system, also known as the major histocompatibility complex (MHC), plays a central role in immune regulation by presenting peptide antigens to lymphocytes, therefore molding the adaptive immune response [8, 11–13]. HLA molecules are critical for peptide binding and T- and B-cell activation. Polymorphisms in HLA molecules are associated with either susceptibility or protection against various infectious and autoimmune diseases by modulating the presentation of self or pathogenic peptides [11–14]. In myocarditis and DCM, specific HLA alleles have been implicated in disease occurrence and progression [15–20]. This review highlights current knowledge of HLA associations with myocarditis and DCM, reviews potential mechanisms of influence, and identifies knowledge gaps and future directions for translating HLA research into clinical care.

## HLA Associations in Myocarditis and DCM

### Specific HLA Associations with Myocarditis and DCM

HLA molecules are divided into two classes. HLA class I molecules (HLA-A, -B, -C) present antigenic peptides to CD8 + T cells, and class II molecules (HLA-DR, HLA-DQ, and HLA-DP) present peptides to CD4 + T helper cells; both classes have been implicated in myocarditis and DCM (Table 1) [8, 11, 12, 21]. In myocarditis, inflammatory cytokines upregulate class I molecules in cardiac myocytes, increasing the presentation of viral and self-peptides to CD8 + T cells [11, 22, 23]. Specific HLA class I alleles influence myocarditis susceptibility in certain situations (e.g., drug-induced myocarditis) [24, 25]. However, most research has focused on HLA class II molecules and CD4 + T helper cells because these molecules are central to orchestrating immune responses [8, 12]. HLA class II molecules typically present cardiac protein fragments to CD4 + T cells [8, 11]. In some cases, class II molecules can be abnormally induced in cardiac cells that do not usually express them, theoretically breaking immune tolerance by directing self-antigens in the heart [22].

**Table 1:** Comparison of HLA class I and class II functions in myocarditis and DCM

Feature	HLA Class I (HLA-A, -B, -C)	HLA Class II (HLA-DR, -DQ, -DP)
Antigen Presentation	Presents peptides (viral or endogenous) to CD8 + cytotoxic T cells [11, 59]	Presents extracellular-derived peptides to CD4 + T helper cells [11, 59]
Cell Types expressing	All nucleated cells including cardiomyocytes [2, 22]	Primarily APCs (dendritic cells, macrophages, B cells); inducible in cardiomyocytes during inflammation [2, 22]
Role in Myocarditis Pathogenesis	Viral infection upregulates HLA-I on cardiac cells; allows direct CD8⁺ T-cell-mediated cytotoxicity [23]	Drives autoimmunity by activating CD4⁺ T cells reactive to cardiac antigens; central in breaking tolerance [12]
Inflammatory Triggers	Interferon-γ can increase HLA-I expression on myocytes [1, 34]	Inflammation can induce de novo HLA-II expression in heart tissue, contributing to autoimmunity [31]
Implication in Autoimmune mechanisms	Indirect—through CD8⁺ T-cell priming and cytotoxicity in viral or drug/vaccine myocarditis [4]	Direct role in autoantigen presentation and loss of self-tolerance, leading to chronic inflammation [8]
Genetic Evidence from GWAS	SNPs (e.g., rs9262636) at 6p21 linked to HLA-I alleles and DCM risk [20]	Same GWAS locus affects expression of class II alleles, reinforcing their role in autoimmune susceptibility [9, 20]

Many candidate-gene studies in multiple populations have shown associations between specific HLA alleles and myocarditis or idiopathic (non-ischemic) DCM (Table 2) [7, 15, 16, 18, 19, 26]. The first evidence appeared in the 1980s and 1990s, when idiopathic DCM was linked to immune dysfunction. Since then, studies have linked HLA-DR4 and HLA-B27 to increased risk for DCM. In one study, DCM patients tested positive for HLA-DR4 at 54%, while healthy controls tested positive at 32% [15], and HLA-B27 was overrepresented in another idiopathic DCM cohort (14.5% of patients vs. 3.3% of controls) [15]. In contrast, HLA-DR3 was underrepresented in DCM patients (odds ratio ~ 0.7), suggesting a protective effect [17]. Other HLA risk alleles have been proposed (e.g., HLA-DQ and -B alleles), but results have not been consistently replicated [18, 19]. Studies with conflicting results demonstrate the genetic heterogeneity in these diseases [7, 26].

**Table 2:** Selected HLA alleles reported in association with myocarditis or DCM

HLA Allele	Reported Association
Class I	
HLA-B27	Elevated frequency in idiopathic DCM (autoimmune DCM) [15]
HLA-A2	Suggestion of higher frequency in DCM (older studies) [15]
Class II	
HLA-DR4	Increased in myocarditis/DCM; risk allele (RR ~ 2); Enriched in idiopathic DCM; linked to anti-β1 receptor Abs; associated with higher risk of progression from myocarditis to DCM [10, 15]
HLA-DR15 (DRB1*15:01/15:03)*	Associated with myocarditis and higher risk of progression to DCM [24, 34]
HLA-DR12 (DRB1*12:01)	Associated with myocarditis/DCM susceptibility [34]
HLA-DR3 (DRB1*03:01)	Underrepresented in DCM (protective effect) [17]
HLA-DQ8 (DQA1*03:01/DQB1*03:02)	Susceptibility to autoimmune myocarditis (seen in transgenic models; prevalent in DR4 haplotype) [29]
HLA-DQB1*03:03	Associated with viral myocarditis without progression (myocarditis with preserved function) [19]
HLA-DQB1*03:01 (+ absence of DQB1*06)	Associated with inflammatory DCM (myocarditis with left ventricular dysfunction) [19]
HLA-DQB1*03:09	Associated with idiopathic DCM in German cohort [18]
HLA-DRB1*14:01	Enriched in COVID-19 mRNA vaccine myocarditis cases [24]
HLA-DRB1*15:03	Enriched in COVID-19 mRNA vaccine myocarditis cases (notably found in some non-European individuals) [24]

Animal models have demonstrated a role for the general HLA region (chromosome 6p21) in DCM susceptibility [20, 27, 28]. The data show that HLA variants control three immune system functions, including immune tolerance, pathogen elimination, and the risk of autoimmune damage, all of which determine whether patients will heal from their injury or develop long-term damage [8, 12, 26].

## Autoimmune Risk Alleles with Shared Mechanisms

HLA alleles known to predispose autoimmunity in other diseases have been linked to myocarditis/DCM, implying shared pathogenic mechanisms [4, 8, 12]. For example, HLA-DR4 and -DQ8 haplotypes, which are highly prevalent in type 1 diabetes, are observed at higher frequencies in both DCM patients and mouse models of autoimmune myocarditis [10, 15, 28, 29]. Furthermore, the HLA-DR3 allele is underexpressed in DCM, suggesting it may confer a protective effect [17]. These patterns support the hypothesis that certain HLA variants can increase the likelihood of viral infection causing a pathological immune response in the heart [4, 12, 14].

## Population Genetic Variations in HLA and Autoimmune Risk Alleles

Associations across ethnic and geographic populations can fluctuate because of differences in HLA allele frequencies [12, 14]. For example, in a Japanese study, viral DCM haplotypes have been identified in the MHC class I/III region, which is susceptible to DCM in patients with an existing hepatitis C infection [30].

## Mechanistic Insights: How HLA Influences Myocarditis and DCM Progression

**Fig. 1:**
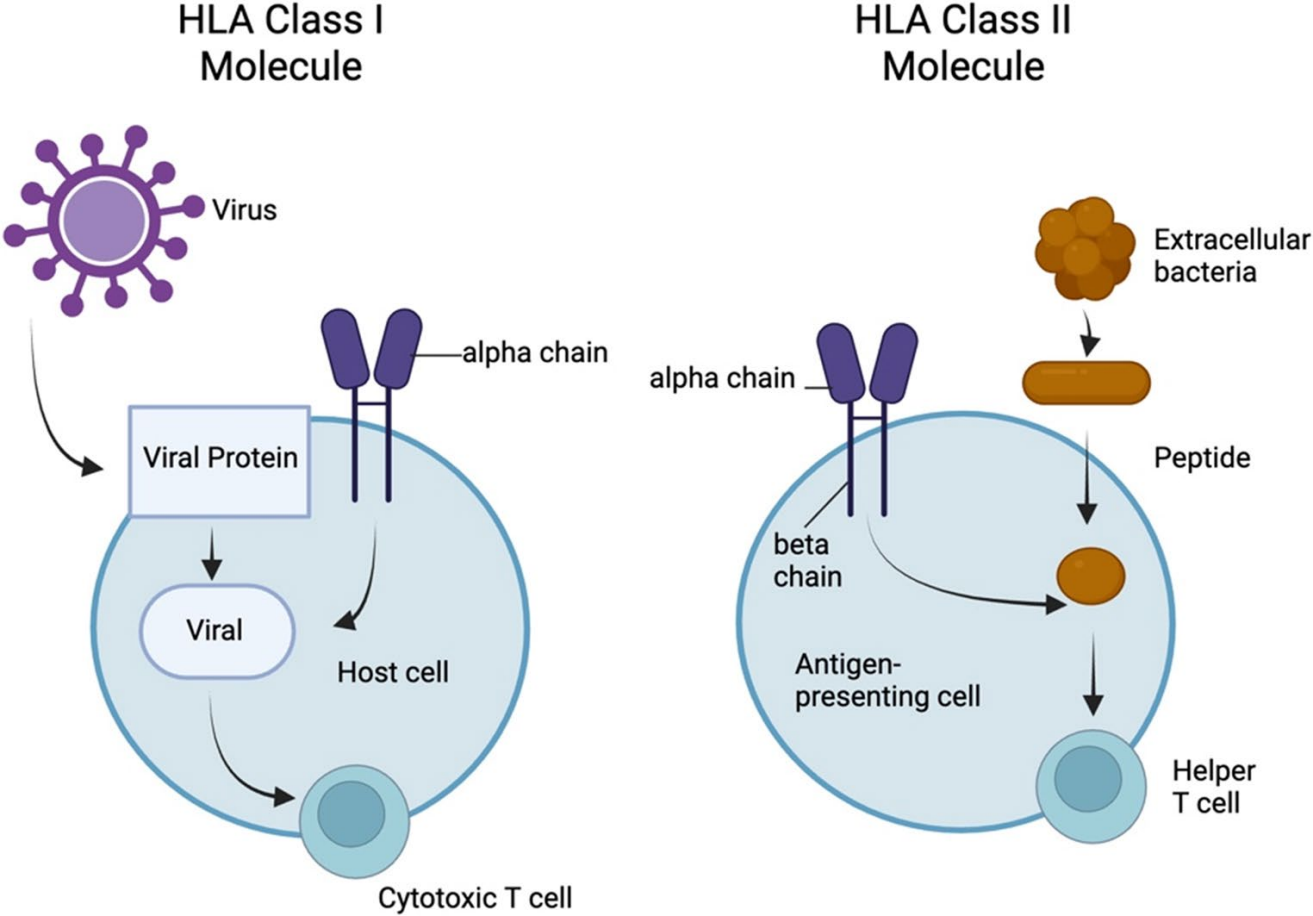
Antigen presentation differences between HLA class I and class II molecules. This diagram contrasts the antigen processing and presentation pathways of HLA Class I and Class II molecules. HLA Class I presents endogenous viral peptides to cytotoxic CD8⁺ T cells, whereas HLA Class II presents exogenous peptides from extracellular bacteria to helper CD4⁺ T cells. The differential antigen presentation underscores the distinct roles of HLA classes in orchestrating immune responses relevant to myocarditis pathogenesis

Myocarditis traditionally occurs in two stages: an initial injury triggers inflammation, followed by an autoimmune response that attacks the heart even after the initial trigger has been cleared [1–3]. HLA molecules play a key role in the autoimmune response through antigen presentation and T-cell activation [8, 11, 12]. This antigen presentation can occur through multiple mechanisms, highlighting how variations in HLA genes can affect myocarditis and DCM (Fig. 1) [4, 8, 12].

## Antigen Presentation and Immune Activation

**Fig. 2:**
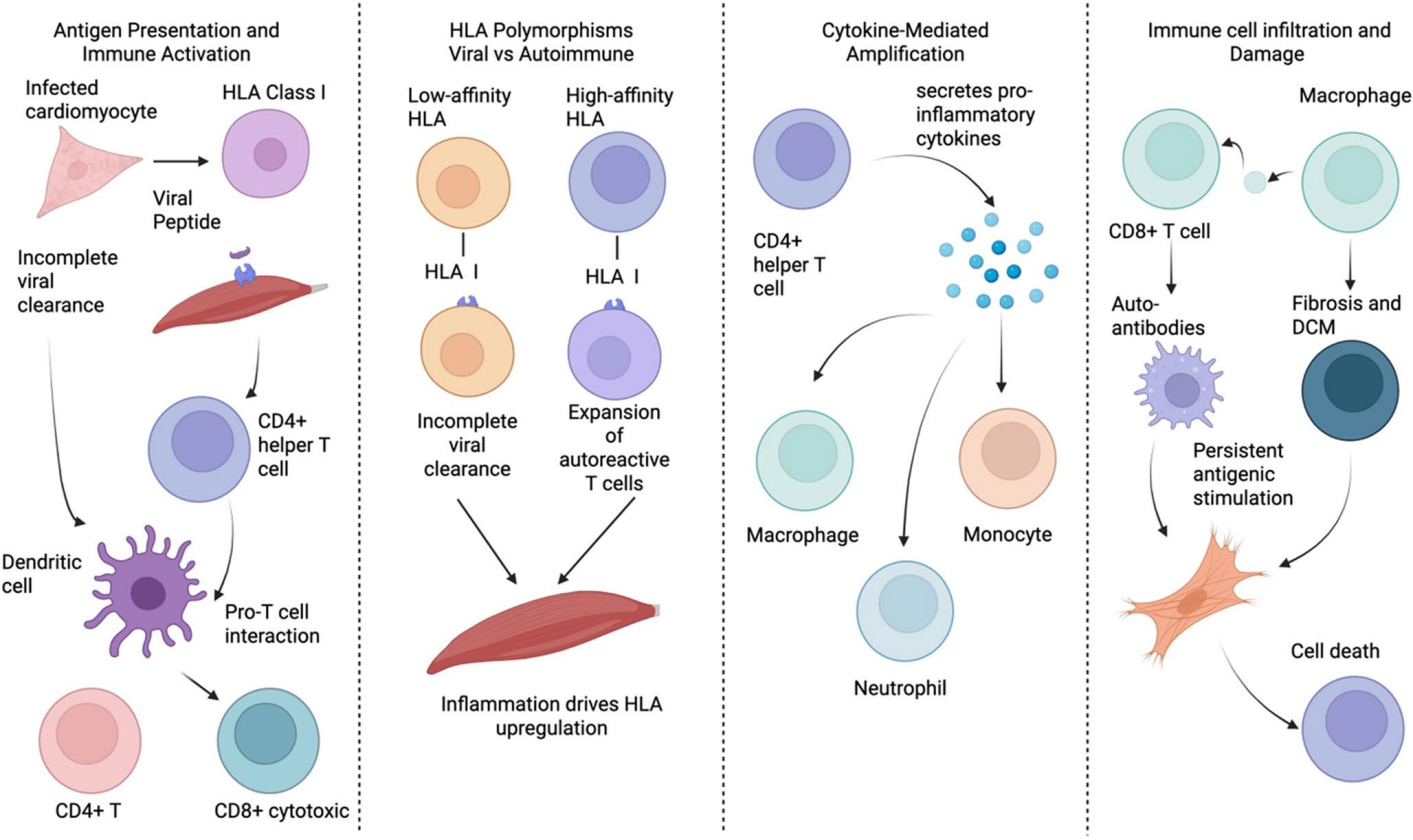
Mechanistic pathways by which HLA polymorphisms influence myocarditis and progression to dilated cardiomyopathy (DCM). HLA-dependent antigen presentation shapes antiviral clearance and autoimmune susceptibility by regulating CD4⁺ and CD8⁺ T-cell activation, cytokine amplification, and immune cell infiltration. High-affinity HLA variants promote persistent inflammation, autoreactive T-cell expansion, and autoantibody production, leading to myocardial injury, fibrosis, and progression to DCM

In acute myocarditis, inflammatory cytokines induce cardiomyocytes to express HLA class I molecules, enabling them to present viral peptides to CD8 + T cells and induce destruction of affected cells [23, 31]. Additionally, professional antigen-presenting cells (APCs) present cardiac antigens to CD4 + helper T cells via HLA class II molecules [8, 11]. Interestingly, some studies have shown that endomyocardial biopsies from patients with myocarditis exhibit an unusual expression of HLA class II in non-APC cells, namely cardiac endothelial cells and cardiomyocytes [7, 22]. This shows that HLA-mediated antigen presentation in inflammatory conditions facilitates both antiviral immune defense and, if deemed necessary, the initiation of autoimmune injury [8, 12, 31] (Fig. 2).

### HLA Polymorphisms and Viral vs. Autoimmune Myocarditis

Many genetic differences in HLA indicate how pathogens are cleared and whether an autoimmune response is triggered [1, 23, 31]. HLA class I molecules bind and present viral peptides efficiently, and have been implicated in the resultant intensity of the CD8 + T cell response [2, 3]. For example, when an allele poorly presents a coxsackievirus epitope, it may cause incomplete viral clearance, prolonging viral infection and myocardial injury [2, 32]. The opposite is also true: an allele that presents a viral peptide more aptly can trigger an immediate response that benefits rapid viral clearance, but can also cause bystander damage and the release of cardiac antigens [2, 3, 32].

HLA class II polymorphisms are significantly implicated in post-viral autoimmunity, as they determine which self-peptides from the heart are recognized by CD4 + T cells [7, 8, 12]. In a genetically susceptible host, HLA alleles that bind self-antigens with high affinity can drive the expansion of autoreactive T cells upon exposure to those antigens [8, 12]. A study shows that different inbred mouse strains with distinct MHC haplotypes exhibit distinct myocarditis outcomes after Coxsackie B3 infection [32, 33]. Some mouse strains can quickly eliminate the virus with only short-term inflammation, whereas other strains develop prolonged myocarditis and DCM-like symptoms, accompanied by autoantibody formation [32]. Additionally, two MHC-associated variations identified in mice show that specific MHC (HLA-equivalent) alleles control the development of viral myocarditis via self-limiting or autoimmune pathways [8, 32].

In one surprising study, transgenic mice that expressed human HLA-DQ8 developed autoimmune myocarditis and cardiomyopathy in the absence of a triggering infection [29]. This demonstrated that an HLA allele optimized to present specific cardiac self-peptides can elicit an immune response against the heart and thereby lower the threshold for developing an autoimmune disease [13, 28, 29]. In clinical settings, this corresponds to situations in which, after an initial infection, patients with “at-risk” HLA alleles are more prone to an extended immune response against cardiac proteins, leading to chronic myocarditis and DCM [1, 7, 8].

If HLA alleles favor the presentation of a certain cardiac myosin peptide, the CD4 + T helper cells specific to that peptide will help coordinate B cells and CD8 + T cells to attack heart tissue [7, 8]. The HLA genotype directly determines how the immune system presents antigens during myocardial inflammation, according to the study results. T cells activate via HLA-peptide complexes, which trigger cytokine production that controls HLA expression and immune cell recruitment in the heart through cytokine-mediated amplification [8, 31, 34].

### Molecular Mimicry and Viral Persistence (Fig. 3)

HLA genes can predispose patients to autoimmune myocarditis through molecular mimicry, an immune response to a foreign antigen (e.g., a virus or bacterium) that cross-reacts with structurally or conformationally similar self-antigens in the heart [4, 8, 35, 36]. If a viral peptide resembles a segment of a cardiac protein and an individual’s HLA allele efficiently presents that viral peptide during infection, activated T cells or antibodies may subsequently recognize the homologous cardiac protein. This process leads to the development of autoimmune disorders [4, 8, 13, 36, 37]. This is the phenomenon behind rheumatic heart disease, where streptococcal M protein elicits antibodies and T cells that cross-react with cardiac myosin and other myocardial proteins [8, 13, 38].

A leading hypothesis in myocarditis is that Coxsackievirus B3 (a common cause of viral myocarditis) may trigger autoimmunity through molecular mimicry mechanisms [3, 13, 32, 33]. Experimental and clinical research have demonstrated that infections with Coxsackievirus can induce the production of T cells and antibodies that recognized cardiac antigens, even though coxsackievirus proteins lacked direct sequence similarity to cardiac myosin [3, 7, 13, 32, 33]. Cardiac myosin heavy chain α is the primary autoantigen driving autoimmune myocarditis via myosin-derived peptides that induce immune responses [4, 7, 33]. Myocarditis-susceptible mouse strains that possess specific MHC haplotypes exhibit T-cell responses targeting specific myosin epitopes, while patients with myocarditis show similar autoreactive immune responses [7, 13, 32, 33]. The presence of HLA alleles that effectively present myosin epitopes increases the likelihood that post-viral immune responses will progress to ongoing autoimmune myocardial injury [4, 13, 33, 36]. Myocarditis-prone HLA-DR and HLA-DQ alleles efficiently present cardiac peptides that share molecular patterns with microbial antigens, according to this accepted framework [13, 27, 33, 36].

Viral persistence provides an additional mechanism through which HLA genotype influences disease progression, as it occurs alongside molecular mimicry [1, 2, 31]. Persistent viral RNA or proteins in the myocardium enable continuous HLA class I-restricted antigen presentation, which keeps CD8⁺ cytotoxic T cells activated, thereby causing ongoing injury to cardiomyocytes and neighboring tissues [1, 2, 23, 31]. Host HLA class I variation may partly determine the efficiency of viral clearance, such that particular alleles promote rapid elimination of cardiotropic viruses, whereas others permit prolonged viral persistence, consequently expanding the window for autoimmune priming [1, 23, 31]. Consequently, HLA alleles that favor strong T-helper responses to cardiac epitopes may amplify antibody-mediated myocardial injury and promote the progression from myocarditis to dilated cardiomyopathy.

Beyond viral triggers, emerging evidence implicates the gut microbiota as an additional source of molecular mimics capable of activating pathogenic cardiac autoimmunity in genetically susceptible hosts [34, 39, 40]. In a seminal study by Gil-Cruz et al., commensal *Bacteroides* species produced peptide mimics structurally similar to cardiac myosin epitopes, resulting in primed autoreactive CD4 + T cells within the gut [39]. Furthermore, disruption of the microbiome with antibiotic treatment prevented myocarditis in a mouse model of spontaneous autoimmune myocarditis, confirming a causal role for microbiota-derived antigenic mimicry in disease initiation [39]. Importantly, similar microbial peptide-specific immune responses have been identified in human myocarditis patients, supporting the clinical relevance of this HLA-microbiome axis [34, 39, 40].

Anti-myosin autoantibodies may also arise during myocarditis and contribute to disease progression. These antibodies can cross-react with β1-adrenergic receptors due to molecular similarity, leading to inappropriate receptor activation, myocardial dysfunction, and adverse ventricular remodeling [6, 26, 41]. Such cross-reactive autoantibodies are detected in a substantial proportion of patients with dilated cardiomyopathy and correlate with disease severity [6, 9, 26, 41]. The generation of these pathogenic autoantibodies is T-cell dependent and requires HLA class II-mediated presentation of cardiac myosin or receptor-derived peptides to CD4 + T helper cells to drive B-cell activation and immunoglobulin class switching [4, 9, 33]. Consequently, HLA alleles that favor strong T-helper responses to cardiac epitopes may amplify antibody-mediated myocardial injury and promote the progression from myocarditis to dilated cardiomyopathy.

## Immune Cell Infiltration and Damage

Once activated, immune effector cells home to the heart [4, 8, 12, 13, 31]. CD4⁺ T cells help macrophages and B cells to congregate while CD8 + T cells directly kill infected or antigen-bearing cardiomyocytes [4, 8, 12, 13, 31]. Monocytes and dendritic cells can ferry cardiac antigens from the site of injury to lymph nodes (sensitizing more T cells) and infiltrate the heart to sustain inflammation [4, 8, 12, 13, 31, 34]. HLA genes indirectly modulate this by forming a pool of autoreactive T cells [4, 13, 42–45]. For instance, if an HLA repertoire fails to eliminate a self-reactive T-cell clone during thymic selection, because the self-peptide is not well presented in the thymus, that clone can later be activated in the periphery when encountering its antigen [8, 13, 42–46]. HLA polymorphisms affect thymic negative selection; some alleles present self-peptides more effectively in the thymus, leading to appropriate deletion of autoreactive T cells, whereas other, less-effectively presenting alleles might allow potentially autoreactive T cells to survive [8, 13, 42–46]. Analysis of T-cell receptors in patients with myocarditis has shown the expansion of T-cell clones specific for heart antigens, demonstrating a failure of tolerance [13, 47–50]. These clones orchestrate myocardial damage by releasing perforin/granzyme (CD8 cells) or activating macrophages (Th1 CD4 cells) and B cells (follicular helper T cells) [4, 8, 12, 13, 31, 47, 48]. The result is myocardial necrosis and apoptosis, which, if extensive and prolonged, heals by fibrosis, leading to the myocardial thinning and dilation characteristic of DCM​ [4, 8, 9, 13, 40]. HLA genes that promote a strong initial immune assault can tip the balance toward adverse remodeling [4, 8, 9, 31, 40]. Conversely, individuals who are genetically predisposed to mount a weaker immune response (perhaps due to “less capable” HLA alleles for those antigens) might clear the infection with minimal autoimmunity and avoid progression to DCM [4, 8, 9, 40].

Overall, HLA influences myocarditis and DCM by determining which cardiac or microbial antigens are presented to the immune system, thereby controlling the specificity of the immune response, and by regulating the intensity and persistence of T cell activation [4, 8, 9, 12, 31, 40]. The triphasic model of myocarditis includes: (1) a trigger causing necrosis and antigen release; (2) a genetically determined, HLA-mediated immune response causing either tolerance or autoimmune attack; and (3) sustained immune attack resulting in chronic inflammation and DCM [4, 8, 9, 40, 51]. When HLA-mediated antigen presentation strongly favors autoimmunity, the progression through phases 2 and 3 is more aggressive [4, 9, 40].

## Knowledge and Transitional Gaps

Despite growing evidence of HLA involvement in myocarditis and DCM, notable shortcomings in current research persist, and translating these data into clinical practice remains difficult [1, 9, 14].

### Limited and Non-Diverse Studies

Many HLA association studies on myocarditis and DCM have been modest in size and limited to single population subsets [1, 10, 14–16]. Early studies often reported weak or inconsistent associations, likely due to small-sample bias [11–13]. Although findings remained significant, most come from populations of European ancestry alone [1, 10, 20]. So far, only weak or modest associations have been reported between idiopathic DCM and certain HLA alleles, suggesting that expanded cohorts are needed to detect consistent genetic signals [15, 16, 18]. HLA allele frequencies vary worldwide; an allele common in one population may be rare elsewhere [20, 30]. For example, HLA-DRB1*15:03 was flagged for post-vaccination myocarditis in an Israeli study but is most frequent in people of African descent, reinforcing that different populations may have different “at-risk” alleles [21, 24].

### Mechanistic Uncertainty

While there are models and hypotheses about how HLA contributes to myocarditis, many details remain unclear [1, 4, 14]. The exact autoantigens driving the immune response in most patients are unknown; cardiac myosin is a prime suspect, but other cardiac proteins could also be targets [4, 7–9, 26, 36]. Linking a specific HLA allele to a peptide antigen is challenging without detailed T-cell epitope mapping, which is more often done in animal models than in patients [8, 9, 28, 29]. As a result, the peptides presented by high-risk HLA alleles in myocarditis are largely unknown [4, 8, 28, 29]. This limits the development of antigen-specific therapies such as tolerizing vaccines [27, 52].

Additionally, the relationship between persistent viral infection and autoimmunity is complex. In some patients, low-level myocardial viral persistence can continuously stimulate HLA-restricted T cells, whereas in others, the virus is cleared but autoimmunity persists [1–3, 31]​. Understanding which scenario is happening is key in dictating treatment (antivirals vs. immunosuppressives); however, this requires expensive and time consuming biopsy analyses (PCR for viral genomes and HLA expression), and the decision is not always clear [1–3]. The heterogeneity of immune responses is another issue: two myocarditis patients with the same HLA type might have very different disease courses, implying that other genes (non-HLA immune genes) as well as exogenous factors (type of virus, timing, microbiome, etc.) strongly modulate outcomes [1–3, 39, 40]. Disentangling HLA effects from this backdrop is difficult. Such data may clarify when and how HLA-driven autoimmunity is self-sustaining.

### Undefined Therapeutic Implications

If certain HLA alleles are associated with a predisposition to, or protection from, myocarditis/DCM, knowing a patient’s HLA alleles could be used to direct therapy. For example, a patient with a “protective” HLA profile might be managed more conservatively. However, this approach is not yet evidence-backed [9, 14]. Another area in need of additional research is the development of therapies that target HLA-peptide-T cell interactions [52, 53]. In myocarditis/DCM, no such antigen-specific therapy is currently available [1, 52]. Intravenous immunoglobulin (IVIG) and steroids are sometimes used in acute myocarditis to broadly modulate the immune response, but results are mixed [2, 3, 31]. There have been few trials of immunoadsorption (to remove cardiac autoantibodies) in DCM patients, which have shown improvements in cardiac function for some, suggesting that immune modulation helps, but it is not targeted by HLA or specific triggers [7, 54–56]. HLA associations might affect only autoimmune-driven cases (e.g., virus-negative or organ-specific autoimmunity). Such approaches are not in clinical use because of safety concerns (Such approaches are not in clinical use because of safety concerns related to significantly hampering the innate and acquired immune systems) [8, 52].

### Complexity of Cardio-Immunology Networks

The immune response in myocarditis involves many cell types - innate immune cells (macrophages, dendritic cells, NK cells), multiple T-cell subsets, B cells, and antibodies [31, 34, 40]. HLA molecules engage with this network primarily during T-cell activation, but downstream effects involve a cascade of signals [31, 34, 40]. As a result, predicting myocarditis/DCM based solely on genetics is challenging [57]. For instance, only a fraction of people with a given “high-risk” HLA allele develop disease, and there is incomplete penetrance [20, 57]. This dilutes the apparent association observed in previous studies, leading to the “weak associations” mentioned earlier [20]. However, these contacts were not well-mapped. Moreover, the same HLA allele might increase susceptibility to different autoimmune outcomes in different patients. This makes it difficult to attribute the specificity to HLA effects.

### Future Directions

#### Large-Scale Genomic and Immunogenomic Studies

There is a pressing need for extensive genomic studies focused on myocarditis and inflammatory cardiomyopathy [14, 20]. Future GWAS or whole-genome sequencing projects with thousands of patients across multiple continents can validate previously reported HLA associations and possibly discover new ones [20]. Importantly, multiethnic cohorts would allow fine-mapping of the HLA region because different populations have different linkage patterns, and a variant that tags a risk allele in one group might be delineated by another group [14]. Researchers should specifically look at HLA imputation or direct HLA sequencing in these studies, since classical HLA alleles may be the true drivers of SNP associations at 6p21 (for example, pinpointing that the SNP in HCG22 is correlated with a particular HLA-DP or HLA-B allele). Additionally, integrating immunogenomic data, such as T-cell receptor sequencing and single-cell RNA sequencing from heart tissue, could reveal which HLA-presented antigens are inciting immune cells in patients with myocarditis [34]. For example, identifying T-cell clones in myocarditis biopsies and testing their recognition (peptide-MHC tetramer studies) could directly link HLA alleles to antigenic targets’ progression to DCM [34, 40].

### Understanding Viral-HLA Interactions

Given that many myocarditis cases are post-viral, future research should delve deeper into how specific virus-HLA combinations affect the disease [3, 23]. Certain viral epitopes may be presented only by particular HLA molecules, thereby influencing viral clearance and immune activation [23, 32]. The example of a hepatitis C-associated DCM and an HLA-linked haplotype demonstrates that infection and host genetics together determine cardiotropic outcomes [30]. Investigating common myocarditis-causing viruses (coxsackieviruses, adenovirus, parvovirus B19, HHV-6) in the context of host HLA could identify high-risk scenarios. Emerging causes, such as COVID-19, including rare cases following mRNA vaccination, should also be studied for HLA associations [21, 24].

Future clinical trials in myocarditis and DCM could stratify or select patients based on immunogenetic profiles, including HLA type [14]. Patients without high-risk HLA alleles may benefit less from immunomodulatory therapies if their disease is primarily viral or non-immune in origin [2]. Experimental models demonstrate that altered peptide ligands can induce antigen-specific tolerance and attenuate autoimmune myocarditis [27, 36]. Therapies targeting T-cell co-stimulation, such as abatacept (CTLA4-Ig), have shown benefits in immune-mediated myocarditis, particularly checkpoint inhibitor-associated disease [25, 58]. B cell-directed therapies and immunoadsorption to remove pathogenic cardiac autoantibodies have demonstrated improvements in cardiac function in subsets of patients with DCM [7].

### HLA-Based Biomarkers for Risk Stratification

As data accumulate, HLA-based polygenic risk scores for myocarditis/DCM may be developed [9]. Such a risk model can prompt closer follow-up and early intervention. In the future, one can envision incorporating the HLA genotype into clinical risk scores along with other markers, such as persistent viral PCR positivity, MRI fibrosis findings, or autoantibody levels. HLA matching is already key in transplantation for alloimmune reasons; now growing evidence supports HLA typing to be key in predicting autoimmune reactions as well [9]. Finally, as biomarkers, circulating immune profiles (such as HLA class II expressing monocytes in blood or soluble HLA molecules) could be explored in conjunction with the HLA genotype to determine if they predict outcomes. Already, case reports using drugs such as [33]or rituximab in refractory myocarditis suggest that targeting the immune system can rescue cardiac function in select cases [40].

## Conclusion

HLA determinants form a crucial part of the myocarditis/DCM puzzle, offering explanations for the observed variability in disease outcomes. HLA genes, as key regulators of antigen presentation, may influence susceptibility to myocarditis or subsequent progression to DCM. HLA class I alleles also contribute, especially in contexts involving viral antigens or drug reactions, by shaping CD8 + T-cell responses. This immune-mediated damage can persist and evolve even after the initial cause (e.g., virus) disappears, culminating in ventricular remodeling and heart failure. The insights gained from HLA associations offer potential for improved risk stratification. Future clinicians could identify high-risk myocarditis patients with unique HLA profiles and treat them more aggressively up front or monitor them more closely for signs of DCM.

**Fig. 3:**
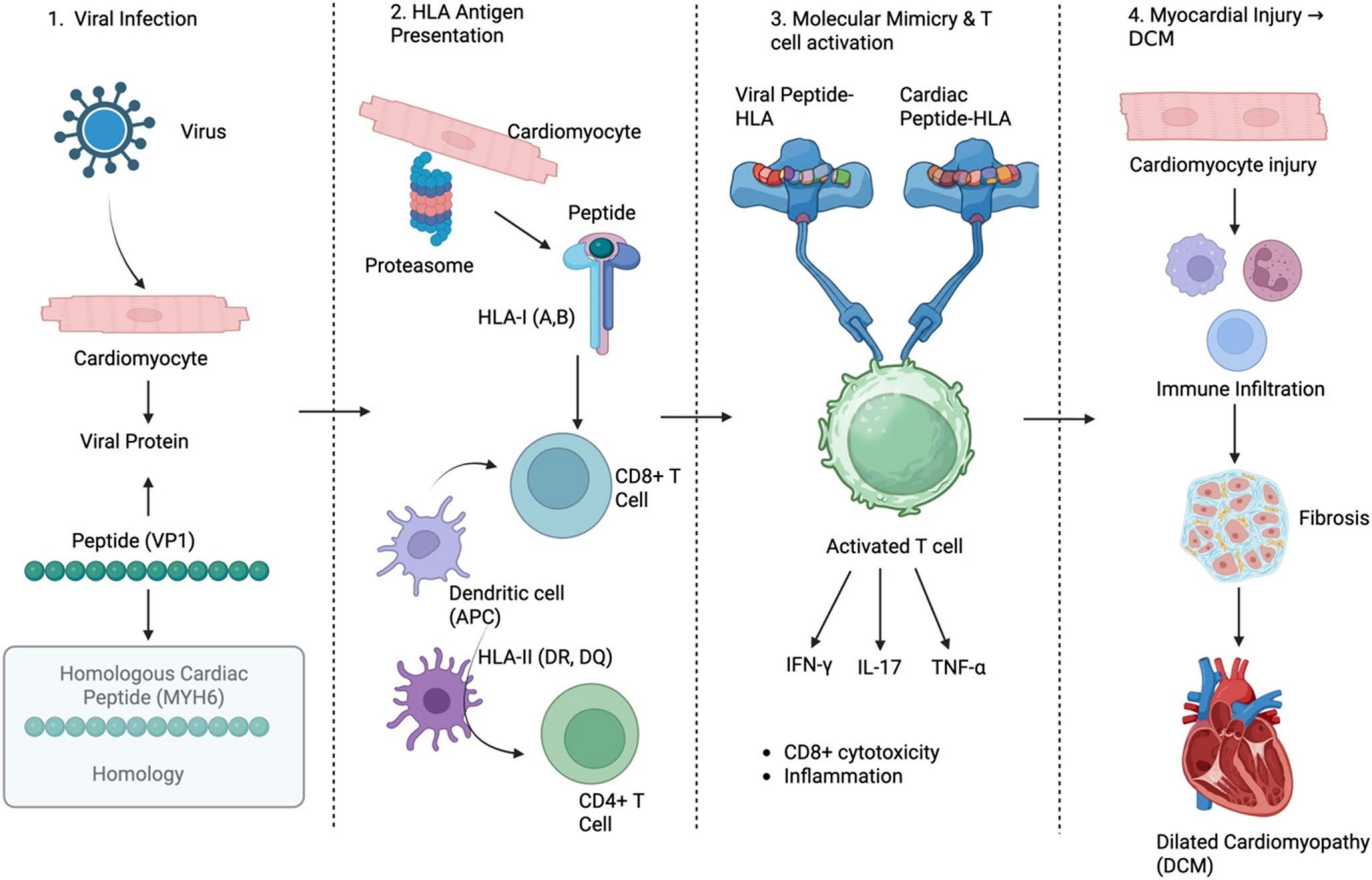
HLA-restricted molecular mimicry in autoimmune myocarditis. Viral infection of cardiomyocytes generates peptides (e.g., VP1) that share structural homology with cardiac self-antigens such as MYH6. Viral and homologous self-peptides are presented via HLA class I and II molecules, activating CD8⁺ and CD4⁺ T cells. Cross-reactive T-cell recognition leads to cytokine production, cytotoxic injury, and sustained inflammation, promoting myocardial damage, fibrosis, and progression to dilated cardiomyopathy (DCM)

## Key References

- Caforio, A.L., et al., Current state of knowledge on aetiology, diagnosis, management, and therapy of myocarditis: a position statement of the European Society of Cardiology Working Group on Myocardial and Pericardial Diseases. Eur Heart J, 2013. 34(33): p. 2636–48, 2648a–2648d.
  - ○ Outlines the comprehensive clinical and immunologic framework of myocarditis, establishing immune-mediated mechanisms as central drivers of disease progression.
- Kindermann, I., et al., Update on myocarditis. J Am Coll Cardiol, 2012. 59(9): p. 779–92.
  - ○ Synthesizes the viral, autoimmune, and inflammatory pathways underlying myocarditis and highlights its progression to dilated cardiomyopathy.
- Caforio, A.L., et al., Novel organ-specific circulating cardiac autoantibodies in dilated cardiomyopathy. J Am Coll Cardiol, 1990. 15(7): p. 1527–34.
  - ○ Provides early evidence linking specific HLA class II alleles with susceptibility to dilated cardiomyopathy.
- Cihakova, D. and N.R. Rose, Pathogenesis of myocarditis and dilated cardiomyopathy. Advances in immunology, 2008. 99: p. 95–114.
  - ○ Conceptualizes myocarditis as a continuum from infection to autoimmunity, forming the mechanistic basis for HLA-driven molecular mimicry.

## Data Availability

No datasets were generated or analysed during the current study.
